# Why Functioning Should Be Used as a Population Health Indicator? A Discussion of a Chilean Population Study

**DOI:** 10.3390/healthcare13131606

**Published:** 2025-07-04

**Authors:** Marina Carvalho Arruda Barreto, Ricardo Cartes-Velásquez, Valeria Campos, Luciana Castaneda, Shamyr Sulyvan Castro

**Affiliations:** 1 Public Health Department, Federal University of Ceará, Fortaleza 60430-140, Brazil; castross@ufc.br; 2 Facultad de Derecho y Ciencias Sociales, Universidad San Sebastián, Concepción 4080870, Chile; cartesvelasquez@gmail.com; 3 Facultad de Ciencias de la Salud, Universidad Autónoma de Chile, Temuco 4810101, Chile; valeriacamposcannobbio@gmail.com; 4 Federal Institute of Education, Science and Technology of Rio de Janeiro, IFRJ, Rio de Janeiro 20260-100, Brazil; luciana.ribeiro@ifrj.edu.br

**Keywords:** International Classification of Functioning Disability and Health, health care, mortality, morbidity, epidemiology

## Abstract

Background/Objectives: Population health problems are among the world’s main concerns. However, mortality and morbidity alone do not fully encompass the health experience of populations. International efforts are underway to verify health experiences using functioning as the third health indicator. The aim of the study was to compare the functioning profile of the Chilean population with mortality and morbidity rates due to neurological, circulatory, respiratory, and musculoskeletal diseases at the regional level. Methods: An ecological study was conducted with the secondary dataset II Estudio Nacional de la Discapacidad (ENDISC) and mortality and hospitalization rates from the Departamento de Estadísticas e Información en Salud. The II-ENDISC was a national household survey, based on the Model Disability Survey, carried out in 2015. Results: The correlation of functioning with morbidity and mortality rates was determined by Spearman’s correlation. The correlation values of the mortality and morbidity coefficients with the performance and disability data were not relevant and significant (*p* < 0.35; *p* > 0.05). This suggests a lack of a linear relationship between these indicators at the regional level. Conclusions: The analysis of the Chilean population revealed that performance values, as an expression of functioning, do not correlate with morbidity or mortality rates. This discrepancy underscores the limitations of relying solely on traditional health indicators to capture the complexity of population health. Our findings support the conceptual value of functioning as a complementary and distinct health indicator, rather than a proxy for morbidity or mortality.

## 1. Introduction

Population health problems are among the world’s main concerns [1]. However, health information has traditionally followed a biomedical model approach, with data collection focusing on issues related to the human body. Nonetheless, in light of the epidemiological transition to non-communicable chronic diseases (NCDs) as the leading causes of health burdens, increasing life expectancy, and population aging, mortality and morbidity cannot cover the whole health experience of populations [2]. It is necessary to understand not only the main causes of death but also the real needs of the population, especially in the context of technological advances that enable people to live with chronic conditions while experiencing varying levels of disability [2,3].

International efforts are underway to verify health experiences using functioning as the third health indicator [4]. This shift in perspective highlights the importance of understanding how health manifests in people’s daily lives. Functioning is the complete lived experience of health: biological and lived health considering health conditions, under consideration of a person’s resources, and in interaction with the environment [4,5].

Health systems require data that reveal aspects of participation and the context in which people live. In the search for information that enables better planning of health care strategies [6], functioning information can be the indicator to offer a perspective that is closest to people’s needs. In line with this trend, a section on functioning has been incorporated into the International Classification of Diseases and Related Health Problems—11 (ICD-11), aiming to measure and classify the impact of health conditions in terms of functioning [7]. More specifically, the ICD-11 integrates the framework of the International Classification of Functioning, Disability and Health (ICF) by providing optional ‘extension codes’ that allow for the detailed classification of a health condition’s impact on a person’s body functions, body structures, activities, and participation.

In addition, providing data on rehabilitation needs is important since the Global Burden of Disease estimates that about 2.4 billion people will require some rehabilitation throughout their lives [3,8]. Furthermore, with about 15% of the global population experiencing severe diseases, information on functioning and disability plays a fundamental role in shaping public policies, social protection, service delivery, and rehabilitation efforts. Such data not only guide effective therapy tailored to the real needs of the population but also contribute to the development of specific policies at the macro, meso, and micro levels [2,9]. Although diagnosis is a relevant aspect, activity and participation limitations play a central role in individual health experiences. Considering the relevance of functioning on a personal level, it is understood that epidemiology can also benefit from obtaining data that enable a collective understanding of the restrictions on population health experiences [4].

The Model Disability Survey (MDS) is an instrument developed by the World Health Organization (WHO) to provide comprehensive information regarding the functioning of the population [10,11]. The MDS is based on the International Classification of Functioning, Disability, and Health (ICF). The main aim of the MDS is to provide estimates of the prevalence of comparable and standardized disabilities between countries; data and information needed to plan interventions, policies, and programs aimed at people with disabilities; and indicators to monitor the implementation of the recommendations of the United Nations Convention on the Rights of Persons with Disabilities [12]. It offers data on disability along a continuum, ranging from no disability to extreme disability, enabling a greater understanding of the population’s degree of functioning and comparing degrees in health conditions [2,13]. Chile and Cambodia have already employed the instrument in their health surveys [14,15]. Within the MDS, “performance” refers to what an individual does in their current environment, while “disability” is conceptualized as the outcome of the interaction between an individual’s health condition and contextual factors, resulting in difficulties in functioning. For example, a person with a mobility impairment may have a low “performance” score in walking, indicating a high level of disability in this domain.

Data on functioning can serve as an indicator of the health status of a population but also as a measure of interventions and service provision, in addition to monitoring the impact of the health system’s output [16]. There are no studies on the complementarity of indicators of mortality, morbidity, and functioning using a population approach. This is a gap that remains undiscussed and unaddressed in the field of research and health management. In addition, rehabilitation professionals traditionally focus on individual clinical activities, underutilizing their experience and know-how, which could also be applied in generating population data for health management at the regional level. This study aimed to compare the Chilean functioning population profile with mortality and morbidity rates that are associated with neurological, circulatory, respiratory, and musculoskeletal diseases. This is intended to show that population data on functioning can provide additional insight beyond that obtained by mortality and morbidity indicators.

## 2. Materials and Methods

This is an ecological study that use two secondary datasets: the II National Disability Survey (*II Estudio Nacional de la Discapacidad*, II ENDISC) and mortality and hospitalization rates from the Health Statistics and Information Department (*Departamento de Estadísticas e Información en Salud*, DEIS).

The II ENDISC was a national household survey, based on the MDS, carried out in 2015 with civilians by the Ministry of Social Development and Family of Chile in order to guide the formulation of public policies on inclusion of persons with disabilities [17]. II-ENDISC used a probabilistic sample of 17.780 people from urban and rural areas of Chile, comprising civilians from 2 to 17 years old and over 17 years old. Sampling process was carried out selecting first the area (urban/rural), resulting in 128 urban strata and 98 rural strata. A second process randomly selected the households into each stratum, and the last selection process was used to randomly choose the interviewee at the residence.

Data collection was carried out from July 2015 to September 2015, and the MDS questionnaire was used. Sociodemographic, health comorbidity, and functioning information were collected. As a main feature, the MDS can highlight the unmet health needs, barriers, and inequalities that people must fight against daily. This article focused on the adult population, aged 18 and over, which resulted from a final sample of 12,265 people, and information about functioning by area of the country. The data are available free of charge at the following link: https://www.senadis.gob.cl/pag/355/1197/ii_estudio_nacional_de_discapacidad (accessed on 2 January 2025) [17,18]

Adult (aged 18 and over) morbidity and hospitalization data from 2015 are publicly available from the DEIS website of the Ministry of Health of Chile [18]. Information about NCDs selected for analysis. This selection was due to the increase in these health conditions and the fact that they are the main causes of impacts on the health of the population at a global level. The variables included in the analysis were performance; disability; and mortality and morbidity rates (circulatory, respiratory, nervous, and muscle and connective tissue).

Performance and disability data were obtained from the II ENDISC databank. As per the MDS methodology, performance “describes what a person does in their real environment” and varies from 0 (best) to 100 (worst score) [19]. Disability is defined as the interaction between a person with a health condition and environmental and personal factors [4], the categories are no disability, mild to moderate, and severe disability. In the context of the MDS, performance and disability scores are derived from self-reported data on a person’s difficulty in various domains of functioning, such as mobility, self-care, and communication. Mortality rates were calculated based on the number of deaths from health conditions in each region: (number of deaths/total population in the area) × 100. Morbidity rates were calculated using the same procedure with the hospitalization data.

Descriptive statistical analysis was performed using frequencies and 95% confidence intervals. The correlation of functioning (general and by disability category) with morbidity and mortality rates was determined by Spearman’s correlation. The study employed a sample design that incorporated stratification and weighting, and, as such, all analyses were conducted using the svy package in Stata 11 (State Corp., College Station, TX, USA) to ensure the appropriate consideration of weights.

## 3. Results

Table 1 and Table 2 describe the mortality, morbidity, and functioning rates of the Chilean population by region.

Table 1 presents data relating to functioning. Average performance values ranged from 27.75 (CI95% 25.24; 30.26) in the Antofogasta Region to 38.61 (CI95% 37.29; 39.93) in the Arica y Parinacota Region. The average of people without disabilities ranged from 27.49 (CI95% 21.15; 33.83) in the Antofogasta Region to 41.62 (CI 95% 39.30; 43.93) in the Las Lagos Region. In the mild to moderate disability range, the average ranged from 47.75 (CI95% 45.62; 49.87) in Valparaiso to 51.78 (CI95% 50.60; 52.62) in the Maule Region. Moreover, in the severe disability range, the average ranged from 54.19 (CI95% 46.98; 61.40) in the Aysen Region to 61.60 (CI 95% 57.77; 65.43) in the Arica y Parinacota Region.

Table 2 describes the mortality and morbidity rates by region of Chile, broken down by circulatory, respiratory, nervous, musculoskeletal, and connective tissue systems. Among the systems, the circulatory system had the highest morbidity rate in all the regions, followed by the respiratory system or musculoskeletal system and connective tissue, depending on the region. Magallanes y La A.C (99.78/10.000) had the highest rate related to morbidity, being related to the circulatory system, and O’Higgins (4.40/10.000) the lowest, being related to the nervous system. In terms of mortality data, the respiratory system demonstrated the highest rates, and there were no data on the musculoskeletal system and connective tissue. La Araucanía (57.1/100,000) had the highest relative mortality rate, being related to the respiratory system, while the region of Aysén (7.3/100,000) had the lowest, referring to the nervous system. A notable finding is the regional discrepancy between functioning and traditional indicators. For instance, the Arica y Parinacota Region showed the highest average performance score (38.61), indicating poorer functioning, while its nervous system mortality rate was low (8.3/100,000). Conversely, La Araucanía had a relatively high mortality rate for the respiratory system (57.1/100,000) but a lower average performance score (31.78) compared to other regions.

Table 3 presents the values found in the correlation analysis between the functioning data and the mortality and morbidity rates. Only the correlation of the respiratory system mortality rate with performance presented a value of *p* < 0.05; however, the correlation was very low, which makes it irrelevant. Therefore, the data indicate that there are no significant correlations between the rates evaluated. These non-significant findings suggest that functioning, as measured by performance and disability scores, does not have a strong linear relationship with morbidity and mortality rates at the regional level, highlighting its potential value as a distinct and complementary health indicator.

## 4. Discussion

In this study, the performance variable was used as an expression of the functioning of the Chilean population. The results show no correlation between performance (general and by disability category) and mortality or morbidity rates.

Among the regions of Chile, those with the best results regarding functioning data were Antofagasta, Valparaíso, and Aysén. Regarding morbidity and mortality, the lowest prevalences were observed in O’Higgins and Aysén, respectively. On the other hand, the regions with the worst functioning values were Arica y Parinacota, Los Lagos, and Maule. Regarding the highest morbidity rates, Magallanes y La A.C. had the highest rate related to the circulatory system, while La Araucanía showed the highest mortality prevalence associated with the respiratory system. These data reinforce the perception that Chilean regions exhibit heterogeneous results in terms of functioning, morbidity, and mortality. These findings suggest that the regions with the highest morbidity or mortality rates do not necessarily correspond to the regions with the worst functioning scores. This variability highlights the need for a deeper analysis to understand the population-level impacts. In the correlation analysis of the data, no statistically significant results were found.

The absence of correlations between the variables suggests a different pattern of functioning (performance) compared to mortality data. While our finding supports the conceptual argument that functioning is a distinct health indicator, we acknowledge that a null result should be interpreted with caution. Alternative explanations for the absence of a strong correlation should be considered. For example, weak associations may be masked by measurement error in either the MDS data or the DEIS statistics. The process of regional aggregation could also obscure individual-level relationships, leading to a diminished correlation at the ecological level. Finally, it is plausible that unmeasured confounders—such as regional socioeconomic status, health system capacity, or environmental factors—could be influencing both sets of indicators independently, thus confounding any direct relationship between them. This nuanced interpretation of our null findings is crucial to avoid overstating the implications of our results.

Some aspects of the relationship between mortality and functioning or disability remain subjects of research, for example, the association between the occurrence of disability and an increased risk of death from all diseases [20]. Difficulty in complex activities and social participation, aspects related to functioning, are also determinants of a higher risk of mortality [21]. The relationship between the occurrence of morbidities and the incidence of disability is already documented in the literature as well [22].

Health assessment, from a biopsychosocial perspective, must include environmental and personal factors and the function and structure of the body to obtain a realistic understanding of the population’s health [4,23]. The literature describes the inclusion of functioning among health indicators, enabling in-depth and coherent knowledge of population health [24].

As Stucki and Bickenbach revealed, functioning is focused on patient-centered outcomes (their health capacity to live a full life), and, as mentioned before, it cannot only be measured with a morbidity indicator [4]. Morbidity does not consider the external factors that can determine what a person can or cannot do, which can even go beyond the scope of a health strategy. Chilean data on performance behave differently from hospitalization (morbidity) and mortality rates. This fact indicates that aspects of the population’s health are not considered when using only morbidity and mortality as health indicators.

Obtaining health information from the population is essential to improve the management of actions and for the successful operation of health systems, assisting in the development of goals and the evaluation of implemented interventions [4], health research being the most comprehensive means of collecting information about functioning and disability [12]. The inclusion of functioning as a health indicator can improve the health strategy. In addition, functioning provides health professionals the possibility of improved analysis of their actions and a greater understanding of the health of the population they serve, aiding in decision-making to develop more equitable health systems and in monitoring the evolution of the results of interventions, as well as enabling the development of health promotion with actions aimed at populations at greater risk of developing disabilities.

It is understood that the use of functioning is becoming increasingly widespread due to the possibilities that this variable can offer. In line with this trend, a section on functioning has been incorporated into ICD-11 to measure and classify the impact of health conditions in terms of functioning [21].

One limitation of this study is that the data were all self-reported. It has been established that self-report is not a very reliable source, but, in national surveys, it is the most viable option. Also, native people may have been excluded from answering the survey as there is no information about whether the interviewers knew the native languages. The study’s results highlight the need for the analysis of functioning findings in other countries, with the inclusion of this variable in health surveys. Additionally, studies should aim to identify the socioeconomic characteristics of the population that have the greatest impact on functioning. As an ecological study, our analysis is subject to ecological fallacy, meaning that the lack of correlation observed at the regional level may not reflect associations at the individual level between functioning and morbidity/mortality. Our reliance on simple bivariate correlations is also a methodological choice that serves our primary research objective but limits our ability to disentangle complex relationships and control for confounding variables. Future studies should address these limitations by exploring stratified analyses and employing more robust analytical techniques, such as multivariate regression, to better understand the complex interplay between functioning, disease burden, and socioeconomic factors.

The population’s performance values (expression of functioning in this study) do not follow the same pattern as the morbidity and mortality indicators. Therefore, it is appropriate to discuss the inclusion of information about functioning as a health indicator. This inclusion will add aspects of health that are not covered by the classic outcomes, increasing, improving, and qualifying health care towards a more equitable approach.

## 5. Conclusions

This study highlights the importance of considering functioning as a health indicator that is distinct from traditional morbidity and mortality metrics. The analysis of the Chilean population revealed that performance values, as an expression of functioning, do not show a strong linear correlation with morbidity or mortality rates at the regional level. This discrepancy underscores the limitations of relying solely on traditional health indicators to capture the complexity of population health. Our findings support the conceptual value of functioning as a complementary indicator, providing unique insights that are not captured by traditional metrics.

## Figures and Tables

**Table 1:** Functioning (performance) of Chilean population according to level of disability by region, II EDISC 2015.

Region	Variables			
		Performance		
		Mean/CI95%		
	Performance–Mean/CI95%	No Disability	Mild to Moderate Disability	Severe Disability
Taparacá	33.76/30.59–36.93	37.61/34.47–40.75	51.39/49.07–53.71	55.72/50.88–60.55
Antofogasta	27.75/25.24–30.26	27.49/21.15–33.83	50.09/48.59–51.59	56.53/49.86–63.20
Atacama	35.70/33.65–37.76	40.97/37.48–44.46	48.72/45.60–51.84	58.29/55.84–60.74
Coquimbo	36.26/34.48–38.04	40.33/38.60–42.07	48.38/45.30–51.46	59.35/55.57–63.14
Valparaíso	33.96/32.73–35.19	36.81/34.19–39.46	47.75/45.62–49.87	58.32/56.24–60.40
O’Higgins	31.76/29.70–33.82	35.92/33.27–38.58	48.95/45.80–52.10	58.67/56.61–60.73
Maule	35.68/34.06–37.30	38.05/31.44–44.66	51.78/50.60–52.64	58.72/56.07–61.36
Biobío	34.69/33.43–35.94	38.96/37.34–40.57	49.66/48.53–50.79	58.97/57.33–60.61
La Araucanía	35.64/33.83–37.45	38.02/34.89–40.57	50.77/48.85–52.69	60.04/57.88–62.20
Los Lagos	37.31/35.67–38.94	41.62/39.30–43.93	48.66/47.89–49.43	58.85/56.85–60.84
Aysén	31.07/28.72–33.43	27.75/20.54–34.97	49.60/47.76–51.44	54.19/46.98–61.40
Magallanes y La A.C	36.97/34.14–39.81	39.37/35.03–43.72	51.62/49.84–53.40	57.21/50.17–64.24
Metropolitana	34.44/33.51–35.38	37.65/36.20–39.09	50.07/48.96–51.19	59.15/57.79–60.50
Los Ríos	36.60/34.56–38.64	37.34/34.29–40.99	50.02/46.96–53.09	56.27/54.62–57.92
Arica y Parinacota	38.61/37.29–39.93	39.29/34.91–43.67	48.12/46.12–50.12	61.60/57.77–65.43

CI95%: confidence intervals.

**Table 2:** Mortality and morbidity rates.

Region	Variables							
	Morbidity Rates				Mortality Rates			
	Nervous System (/100,000)	Circulatory System (/10,000)	Respiratory System (/10,000)	Musculoskeletal System and Connective Tissue (/100,000)	Nervous System (/100,000)	Circulatory System (/10,000)	Respiratory System (/100,000)	Musculoskeletal System and Connective Tissue (/100,000)
Taparacá	7.54	57.81	25.41	28.89	11.5	11.34	27.9	-
Antofogasta	7.77	59.89	38.56	43.92	14.4	11.54	32.2	-
Atacama	7.55	57.60	32.06	20.86	12.1	12.70	37.13	-
Coquimbo	6.36	50.94	29.36	24.77	15.0	13.51	40.8	-
Valparaíso	12.9	87.03	50.92	45.71	20.2	19.25	53.0	-
O’Higgins	4.40	61.31	43.33	35.33	14.0	16.25	53.0	-
Maule	8.14	68.29	49.26	28.74	17.3	17.37	56.9	-
Biobío	12.38	78.13	60.59	39.85	18.0	16.19	46.1	-
La Araucanía	12.35	90.27	69.96	24.60	14.6	16.27	57.1	-
Los Lagos	11.55	80.04	53.34	42.26	21.2	14.73	52.3	-
Aysén	14.30	77.45	54.27	34.24	7.3	11.44	47.0	-
Magallanes y La A.C	28.36	99.78	67.41	80.89	15.1	18.09	34.0	-
Metropolitana	11.58	62.94	41.15	42.02	16.3	15.07	45.6	-
Los Ríos	14.93	93.11	71.92	41.36	23.7	16.71	52.6	-

**Table 3:** Correlation values of the mortality and morbidity coefficients with the functioning data, 2015.

Health Condition	Performance	Disability		
		None	Mild to Moderate	Severe
	Morbidity			
Nervous system	−0.1577	0.3429	−0.1639	−0.1126
Circulatory system	−0.1408	0.0560	−0.1593	−0.0834
Respiratory system	−0.1872 *	0.0060	−0.1110	−0.0908
Musculoskeletal system and connective tissue	−0.1023	0.1012	−0.0717	0.0396
	Mortality			
Nervous system	−0.0357	0.2954	−0.1900	0.0093
Circulatory system	−0.1003	0.1072	−0.0365	−0.1732
Respiratory system	−0.1115	−0.0165	−0.0662	−0.1121
Musculoskeletal system and connective tissue ^#^	-	-	-	-

* *p* < 0.05; # there was no mortality due to musculoskeletal diseases.

## Data Availability

The data associated with the paper are available in the https://www.senadis.gob.cl/pag/356/1625/base_de_datos repository (accessed on 2 January 2025).
